# Myocardial Infarction Triggered by Marijuana Use

**DOI:** 10.1016/j.jaccas.2024.103202

**Published:** 2025-02-12

**Authors:** Vaibhav Sharma, Anjali Bhardwaj, Akshat Sahai, Surender Singh, Shariq Shamim

**Affiliations:** a Department of Cardiovascular Disease, St. Louis Heart and Vascular, St. Louis, Missouri, USA; b Vassar Brothers Medical Center, Poughkeepsie, New York, USA

**Keywords:** coronary angiography, echocardiography, electrocardiography, intravascular ultrasound, myocardial infarction, percutaneous coronary intervention

## Abstract

Chronic marijuana use has been increasingly linked to cardiovascular events, though reports of acute myocardial infarction in young adults without traditional risk factors remain limited. A 24-year-old man with a 9-year history of daily marijuana use presented with acute chest pain. Emergency evaluation revealed anterior ST-segment elevation myocardial infarction with 100% proximal left anterior descending coronary artery occlusion, treated successfully with percutaneous intervention. Intravascular ultrasound showed isolated plaque rupture in an otherwise normal coronary tree. This case highlights marijuana’s potential role in precipitating acute coronary events in young adults without traditional risk factors, supported by intravascular imaging findings.

## Graphical Abstract

**Figure:**
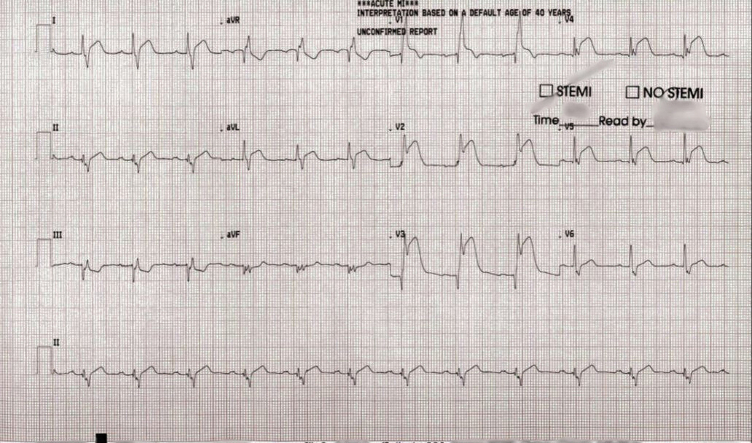

## History of Presentation

A 24-year-old man with no significant medical history presented to the emergency department with a chief symptom of sudden-onset severe sharp chest pain radiating to the left arm, persisting for a duration of approximately 40 minutes. The episode was accompanied by associated symptoms of nausea, vomiting, and significant anxiety. The patient reported a history of intermittent episodes of chest discomfort over the preceding 2 months frequently accompanied by nausea and vomiting, although none was as severe as the current presentation. Notably, the patient reported a transient improvement in nausea and vomiting with hot-water showers.

**Take-Home Messages**

• Long-term cannabis use is a potential etiologic factor in acute coronary syndrome, especially in young patients without traditional risk factors.
• A comprehensive toxicologic screening, including tests for cannabinoids, could provide valuable insights into the underlying causes of acute coronary syndrome.
• Clinicians should consider marijuana use when evaluating young patients with chest pain, even in the absence of traditional cardiovascular risk factors.

Upon presentation, the patient was hypotensive, with blood pressure of 78/52 mm Hg. His pulse was regular, with a tachycardic rate of 140 beats/min, and characterized by a low volume but normal character, without any significant delays on pulse examination. His oxygen saturation was 97% on room air, and his body temperature was afebrile at 37.8 °C. Physical examination revealed the presence of grade 3 pitting edema in the lower extremities, along with distended internal jugular veins, implying elevated central venous pressure.

## Past History

The patient had no significant medical history. He denied any history of acute coronary events or history of stroke in his family. However, he disclosed a 9-year history of daily marijuana use in the form of smoke, while denying the use of alcohol and tobacco in any forms. He also denied the use of any other psychotropic substances. His family history was noncontributory, and he reported no prior hospital admissions.

## Differential Diagnosis

When considering the patient’s presentation, several potential diagnoses were contemplated. One primary consideration was marijuana-induced coronary vasospasm, which could explain the acute cardiovascular event. Given the patient’s young age, spontaneous coronary artery dissection was also a critical differential diagnosis to explore. Additionally, the possibility of premature atherosclerosis was examined, though the patient’s age and lack of traditional risk factors made this diagnosis less likely.

## Investigations

**Figure 1:**
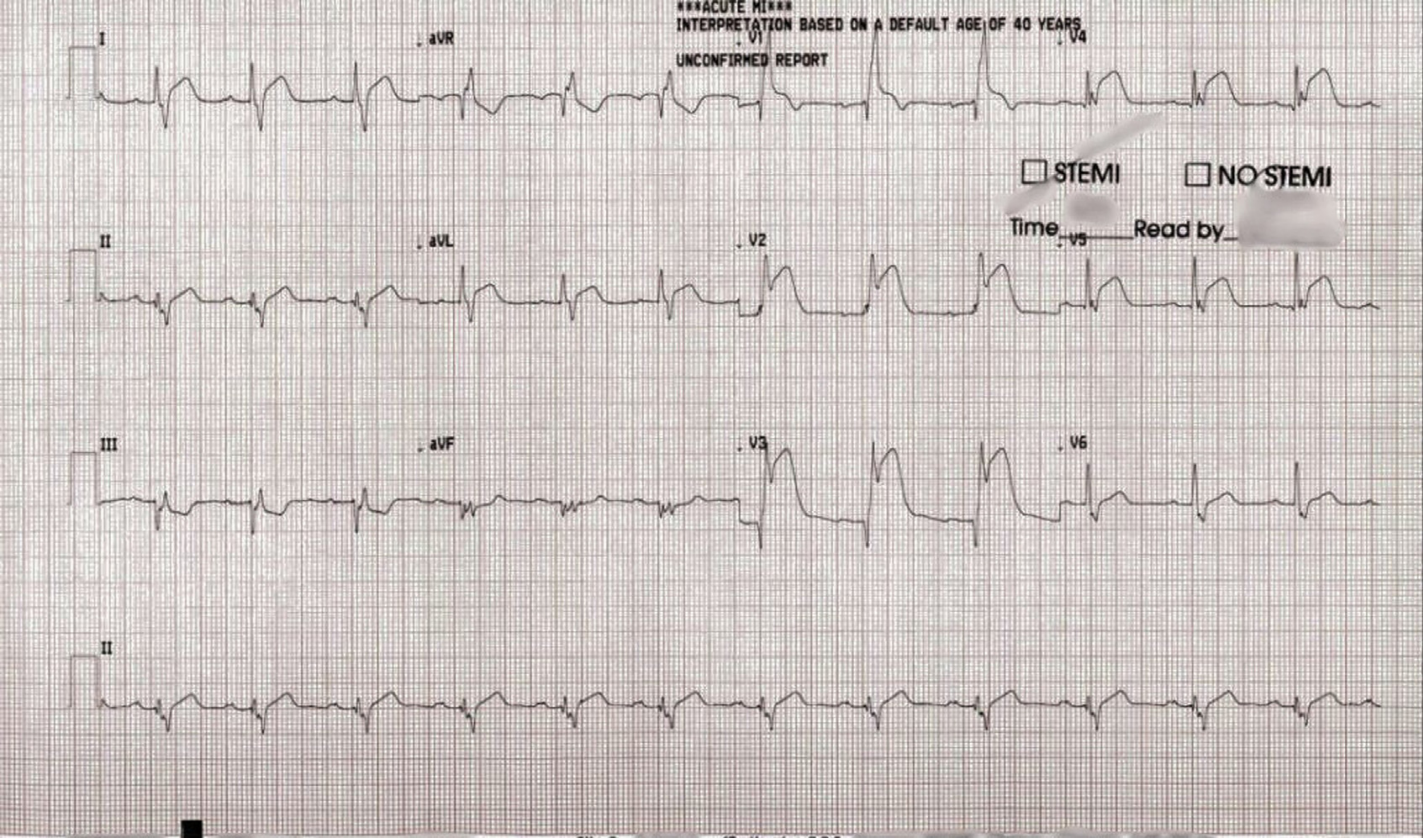
Emergent Electrocardiography Revealed Right Bundle Branch Block and Significant ST-Segment Elevations in the Anterior Leads (V_2_-V_5_), Indicating Acute Myocardial Infarction in the Left Anterior Descending Coronary Artery Territory STEMI = ST-segment elevation myocardial infarction.

Electrocardiography revealed a right bundle branch block along with significant ST-segment elevations in the anterior leads (V_2_-V_5_), indicative of an acute myocardial infarction in the territory of the left anterior descending coronary artery (LAD) (Figure 1). Laboratory investigations revealed markedly elevated troponin I of 249.7 ng/L (normal range: <40 ng/L), while other serologic markers, including lipid profile and inflammatory markers, were within normal limits. Transthoracic echocardiography revealed significant left ventricular dilation with apical akinesis and a reduced ejection fraction of 30% to 35% (Video 1). Urine toxicology conducted in the emergency department highlighted results positive for 11-hydroxy-tetrahydrocannabinol, the active metabolite of marijuana.

## Management

**Figure 2:**
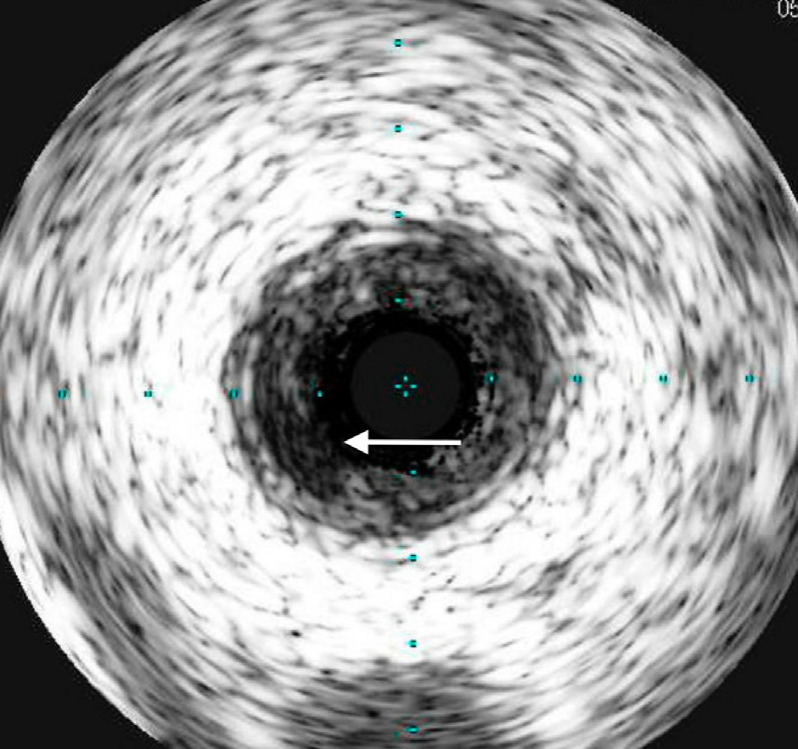
Intravascular Ultrasound Intravascular ultrasound demonstrated a left anterior descending coronary artery free of any atherosclerosis except a very short segment, approximately 10 mm long, in the proximal segment with ruptured plaque (white arrow).

Coronary angiography was conducted through right radial artery access, revealing a totally occluded proximal segment of the LAD (Video 2). Flow was restored with a 2.5 balloon, and subsequently intravascular ultrasound was performed, demonstrating that the LAD was free of any atherosclerosis except a very short segment, approximately 10 mm in length, in the proximal segment with ruptured plaque (Figure 2). Subsequently, a 3.5 × 16 mm Synergy drug-eluting stent (Boston Scientific) was deployed, without any residual stenosis in the treated segment and TIMI flow grade 3 (Video 3).

Postprocedural management included the initiation of guideline-directed medical therapy, comprising dual antiplatelet therapy (aspirin and clopidogrel), high-intensity statin therapy (atorvastatin), beta-blockade (metoprolol succinate), and angiotensin-converting enzyme inhibition (enalapril). Despite timely revascularization, there was a very high increase in high-sensitivity troponins to >10,000 ng/L (normal range: <14 ng/L), consistent with a large infarction. Because of the occurrence of acute coronary syndrome in the presence of a severely depressed left ventricular ejection fraction, the patient was discharged with a wearable cardioverter-defibrillator to mitigate the risk for sudden cardiac death secondary to ventricular arrhythmia.

## Outcome and Follow-Up

At 1-month follow-up, the patient remained symptom free. Cardiac magnetic resonance imaging was performed, demonstrating a nontransmural infarction of LAD distribution with an ejection fraction of 40%. The wearable cardioverter-defibrillator was discontinued, and the patient stopped smoking marijuana.

## Discussion

The association between cannabis use and cardiovascular events has gained increasing attention in recent literature. Cannabis intoxication is mediated through the activation of G protein–coupled cannabinoid receptors, primarily CB1 and CB2.^1^ Although CB1 receptors are found predominantly in the central nervous system and modulate neurotransmitter release, CB2 receptors are located primarily in immune tissues and play a role in immune response regulation.^1^^,^^2^

Acute cannabis intoxication typically manifests with tachycardia, hypertension, tachypnea, conjunctival injection, and xerostomia.^3^ However, more severe acute adverse reactions have been documented across various organ systems. Pulmonary complications include exacerbation of poorly controlled asthma and spontaneous pneumothorax, the latter potentially associated with prolonged breath holding during cannabis inhalation.^4^^,^^5^ The acute cardiovascular manifestations of cannabis use, particularly its potential to precipitate myocardial infarction, present a more immediate risk profile compared with the gradual accumulation of metabolic and inflammatory sequelae. The pathophysiological mechanism primarily involves cannabis-induced alterations in coronary vasodynamics and chronotropic effects, which may culminate in myocardial ischemia and subsequent infarction in susceptible individuals.^6^

Of particular concern are the cardiovascular sequelae of cannabis use. The groundbreaking epidemiologic evidence establishing cannabis as a potential risk factor for atherosclerotic cardiovascular disease emerged from Mahtta et al’s^7^ cross-sectional analysis, which used data from the U.S. Department of Veterans Affairs health care system database in conjunction with the VITAL (Veterans With Premature Atherosclerosis) registry. A recent large-scale, population-based cross-sectional study by Jeffers et al^1^ revealed a significant association between daily cannabis use and myocardial infarction. Interestingly, the study also noted an increased risk for stroke among cannabis users who abstained from tobacco use.

A comprehensive literature review by Chetty et al^8^ highlighted the atypical demographic profile of patients presenting with cannabis-induced myocardial ischemia. These individuals were notably younger than the average patient with myocardial infarction and often lacked traditional coronary risk factors such as hypertension, hyperlipidemia, or diabetes.

Although a definitive causal relationship between cannabis use and myocardial ischemia has not been established, mounting evidence suggests a temporal association.^9^^,^^10^ This case presentation exemplifies an increasingly recognized phenomenon of potentially life-threatening myocardial infarction in young adults with chronic cannabis use. The rising incidence of such cases, coupled with the global trend toward cannabis legalization, underscores the urgent need for further research into the cardiovascular implications of cannabis use.

With this case, we highlight the importance of maintaining a high index of suspicion for coronary events in young adults with histories of long-term cannabis use, even in the absence of traditional cardiovascular risk factors. It also emphasizes the need for comprehensive substance use history taking in the evaluation of young patients presenting with acute coronary syndromes.

## Conclusions

Despite the widespread perception of marijuana as a relatively safe substance, emerging evidence suggests that long-term use can lead to severe cardiovascular events, including ST-segment elevation myocardial infarction. This case emphasizes the growing recognition of marijuana as a significant risk factor for myocardial infarction, particularly in young patients without traditional cardiovascular risk factors. The patient’s presentation, with no other identifiable risk factors apart from long-term marijuana use, highlights the importance of considering marijuana as a potential risk factor to myocardial ischemia in young individuals.

Clinicians should maintain a high index of suspicion for myocardial infarction in young patients presenting with chest pain and histories of marijuana use, even in the absence of other cardiovascular risk factors. Incorporating urine drug screening and considering marijuana use as a predisposing factor in acute myocardial events could improve diagnosis and management in this population. As legalization and consumption of marijuana continue to rise, there is an urgent need for further research to elucidate the causal pathways, educate the public on the potential cardiovascular risks, and guide the clinical approach to marijuana-associated cardiac events.

## Funding Support and Author Disclosures

The authors have reported that they have no relationships relevant to the contents of this paper to disclose.

## Appendix

Video 1 Transthoracic Echocardiography Revealed Significant Left Ventricular Dilation With Apical Akinesis and Reduced Ejection Fraction of 30% to 35%

Video 2 Coronary Angiography Revealed a Totally Occluded Proximal Segment of the Left Anterior Descending Coronary Artery

Video 3 Postprocedural Coronary Angiography Revealed a Patent Left Anterior Descending Coronary Artery
